# Popular Science—A Proposed New Department

**Published:** 1881-02

**Authors:** 


POPULAR SCIENCE—A PROPOSED NEW DEPARTMENT.—We are glad to announce to our readers, that with the April No. of the current volume, we will increase the pages of the INDEPENDENT PRACTITIONER, by adding a new department devoted to *Popular Science*. This feature in Medical Journalism, we believe to be a good one, as there are so many scientific publications that it is impossible for the busy practitioner to read all of them. Our object will be, to present to our readers in an intelligent and concise way, the most useful popular scientific subjects. We will probably add a celebrated scientist to our editorial staff.

All those who send in their annual subscription for the current year before the 31st of March, will receive the Journal at our published rates, viz.: $2. After that date the price will be raised to ($3.) three dollars for this year.

We are so much encouraged, that we are warranted in taking this step, and sincerely hope that it will prove acceptable to all.

We invite short and terse articles for this new department, as well as on medicine and its specialties.

